# Transient adhesion in a non-fully detached contact

**DOI:** 10.1038/s41598-018-24587-6

**Published:** 2018-04-18

**Authors:** Zheyu Liu, Hongyu Lu, Yelong Zheng, Dashuai Tao, Yonggang Meng, Yu Tian

**Affiliations:** The State Key Laboratory of Tribology, Department of Mechanical Engineering, Tsinghua University, 100084 Beijing, China

## Abstract

Continuous approaching and detaching displacement usually occurs in an adhesion test. Here, we found a transient adhesion force at the end of a non-fully detached contact. This force occurred when the nominal detaching displacement was less than the traditional quasi-static theory predicted zero force point. The transient adhesion force was ascribed to interfacial adhesion hysteresis, which was caused by the cracking process of the contact and the deformation competition between the sphere and supporting spring. Results indicated that the testing of adhesion can be significantly affected by different combinations of stiffnesses of the contact objects and the supporting spring cantilever. This combination should be carefully designed in an adhesion test. All these results enabled increased understanding of the nature of adhesion and can guide the design of adhesive actuators.

## Introduction

Adhesion widely exists in nature and greatly affects the activities of humans, creatures, and machines. As an example of exceptional control of adhesion, geckos can use setae on their toes to reliably adhere to various walls and ceilings^1^. As an invented product, pressure sensitive adhesives have been widely used in medical treatment and the daily lives of people^2^. Therefore, the study of adhesion has long been an interest for both scientists and engineers. Various contact models have been developed to describe the relationship between the applied load, surface energy, and the elastic deformation in a sphere/sphere contact^3–6^. Hertz contact theory described the pure elastic deformation of a sphere under an external load^3^. Johnson–Kendall–Roberts (JKR) theory considered the internal surface force^4^. Dejaguin–Muller–Toporov (DMT) theory considered the energy of the non-contact adhesion forces acting surrounding the contact area^5^. The two theories were subsequently integrated into the Maugis–Dugdale (MD) theory to incorporate the two extreme situations of material with compliant large radius and rigid small radius^6,7^. Furthermore, for polymers, such as polydimethylsiloxane (PDMS) and polyurethane (PU), the adhesion was strongly related with the detaching velocity^8^. The crack propagation was also introduced into the detaching procedure to calculate the adhesion between viscoelastic solids^9–12^. In addition to the traditional researches on adhesion phenomena and theories^3–6,13,14^, many researchers studied high-performance biomimetic dry and wet adhesive surfaces^15–22^.

In a theoretical analysis of adhesion, the force-displacement relationship is one of the key factors needed to be disclosed^23–34^. In a typical adhesion test, the experiment was usually carried out as follows. One of the adhesion surfaces was usually fixed on a spring cantilever. First, the sphere was brought into contact with a rigid substrate and continuously loaded up to a pre-set value. After dwelling for a certain time, the sphere/plate contact was continuously separated. During the detaching, the repulsive force between the sphere and the plate gradually changed to be attractive and then suddenly dropped to zero after the maximum attractive force. The peak of attractive force (i.e., adhesion force) is usually used as the adhesive strength between the two surfaces.

A typical adhesion test analysis usually only considers the deformation of the sphere. However, in a real adhesion test, the loading is usually conducted via a cantilever, whose deformation is generally not discussed. In fact, in one former adhesion test between a gecko setal array and a glass slide, the stiffness of the cantilever in the adhesion test can significantly affect the tested adhesion force value; alternatively, no adhesion was tested^35^. In a detaching process, the adhesion force usually appears when the unloading displacement is near or over the nominal loading displacement. In this study, the unloading displacement was divided into two steps. A transient adhesion force was observed when the unloading displacement did not exceed the theoretical critical zero force point. The phenomenon was ascribed to the interfacial viscoelasticity of the contact and the deformation compatibility between the sphere and the cantilever. These results can provide additional insights on the comprehension of the tested adhesion, and can guide the selection of cantilever spring stiffness and a proper design of adhesion test procedure.

## Methods and Materials

### Adhesion test system

**Figure 1:**
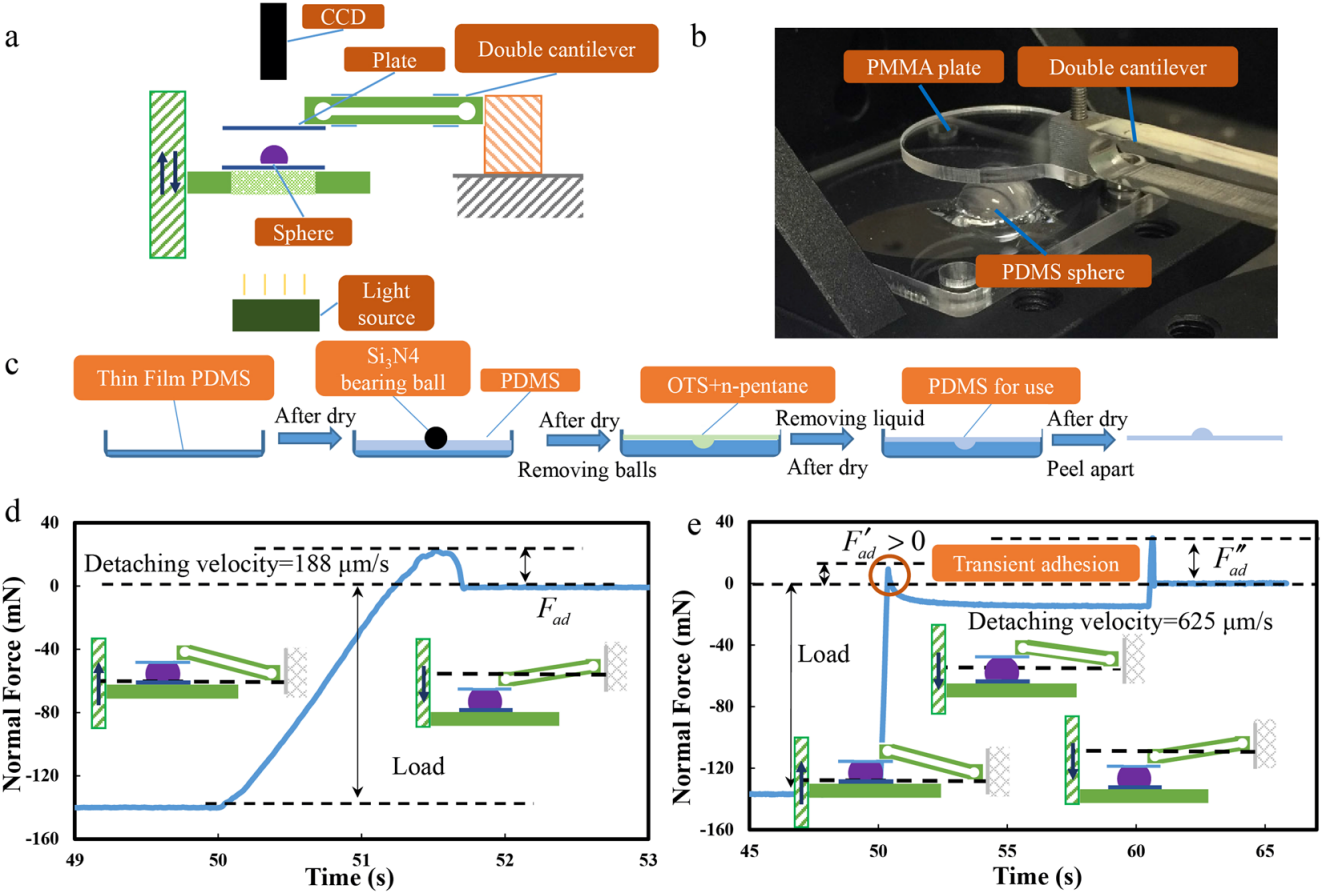
Sketch of the adhesion test system and typical test curves. (**a**) Diagram of the test system; (**b**) Oblique view of the sphere/plate contact; (**c**) Manufacturing procedure of the PDMS sphere; (**d**) Typical force curve of an one-step detachment (a loading displacement *L*_0_ and detaching displacement *L*′_0_ > *L*_0_); (**e**) Typical force curve of a two-step detachment showing an transient adhesion, the first detaching *L*_1_ and the second detaching *L*_2_.

The adhesion test system was sketched in Fig. 1. A double-cantilever glued with strain gauges was used for measuring the normal forces (Fig. 1a,b). The strain gauge signal was processed by an amplifier (BSFY-1, Shijiazhuang Bufson Instrument Technology Co., Ltd.), and recorded at a frequency of 1000 Hz (USB-6002 data acquisition card, National Instrument Co., Ltd.). The movement of the lower plate (glued with a sphere) was driven by a one-dimensional linear stage (TSA30-C, Beijing Zolix Instruments Co., Ltd.) with a resolution of 0.6 μm, which was monitored with a linear encoder (KA-500, Guangdong Sino Grating Digital Display Co., Ltd.).

### Sphere manufacturing

Smooth polymer spheres used in this study were made by reversely molding Si_3_N_4_ ceramic balls (diameter: 4.8 and 11.1 mm, respectively, precision level: G5, *Ra* = 0.014 μm, Shanghai Bujin Advanced Ceramics Co., Ltd.) with PDMS (Sylgard 184, Dow Corning Co., Ltd.) with weight ratios of component A to component B of 20:1, 10:1 and 5:1 (Fig. 1c). Samples were cured in an oven for 2 h at 65 °C. The plate used in the test was made from polymethylmethacrylate (PMMA, *Ra* = 3.58 nm). The plate was replaced by a new one after a serial of tests was finished. The experiment was carried out at room temperature and a humidity of 40%–60%.

### Experiment procedure

In a one-step detachment (OSD), the sphere was loaded on the plate with a loading displacement *L*_0_ = 200 μm and the velocity of 625 μm /s. After 40 s, the sphere was fully separated at the set velocity. A typical result of OSD was shown in Fig. 1d. In a two-step detachment (TSD), the sphere was only separated part of the total loading displacement *L*_1_. Then, after 10 s, a detaching of the rest displacement *L*_2_ was conducted, thereby ensuring a full separation. A typical result of TSD was shown in Fig. 1e. The maximum attractive detaching force in OSD was marked as *F*_ad_. In TSD, the first detaching force peak was marked as *F′*_ad_ and the second was *F″*_ad_. The force curve in OSD is different from that of TSD. The *F′*_ad_ point in TSD was a sharp needle-like peak, whereas the *F*_ad_ peak and *F″*_ad_ peak were parabolic. The detaching velocity of the stage was discrete around the peak *F′*_ad_ in TSD, leading to a needle-like force curve. A continuous separation in OSD resulted in a parabolic peak.

### Determination of the contact radius

Before the experiments, the position of the camera (MQ013MG-ON, XIMEA GmbH) was fixed after the focusing. And the plane light source was placed in a proper position so that the edge of the contact radius can be obviously distinguished. The coordinates of the center of the contact area were recorded before the tests. The change of the contact area during the experiments was recorded by the camera with 500 frames per second. Then the video was converted into a series of pictures frame by frame. These pictures was processed in Halcon HDevelop (MVTec Software GmbH) to identify the difference of the gray level near the edge of the contact area to pick up the coordinates of the boundary point. The contact radius as a function of time was obtained by calculating the relative distance between the center and the boundary point.

### Data availability statement

All the data of manuscript data is available.

## Results and Discussions

### Transient adhesion

**Figure 2:**
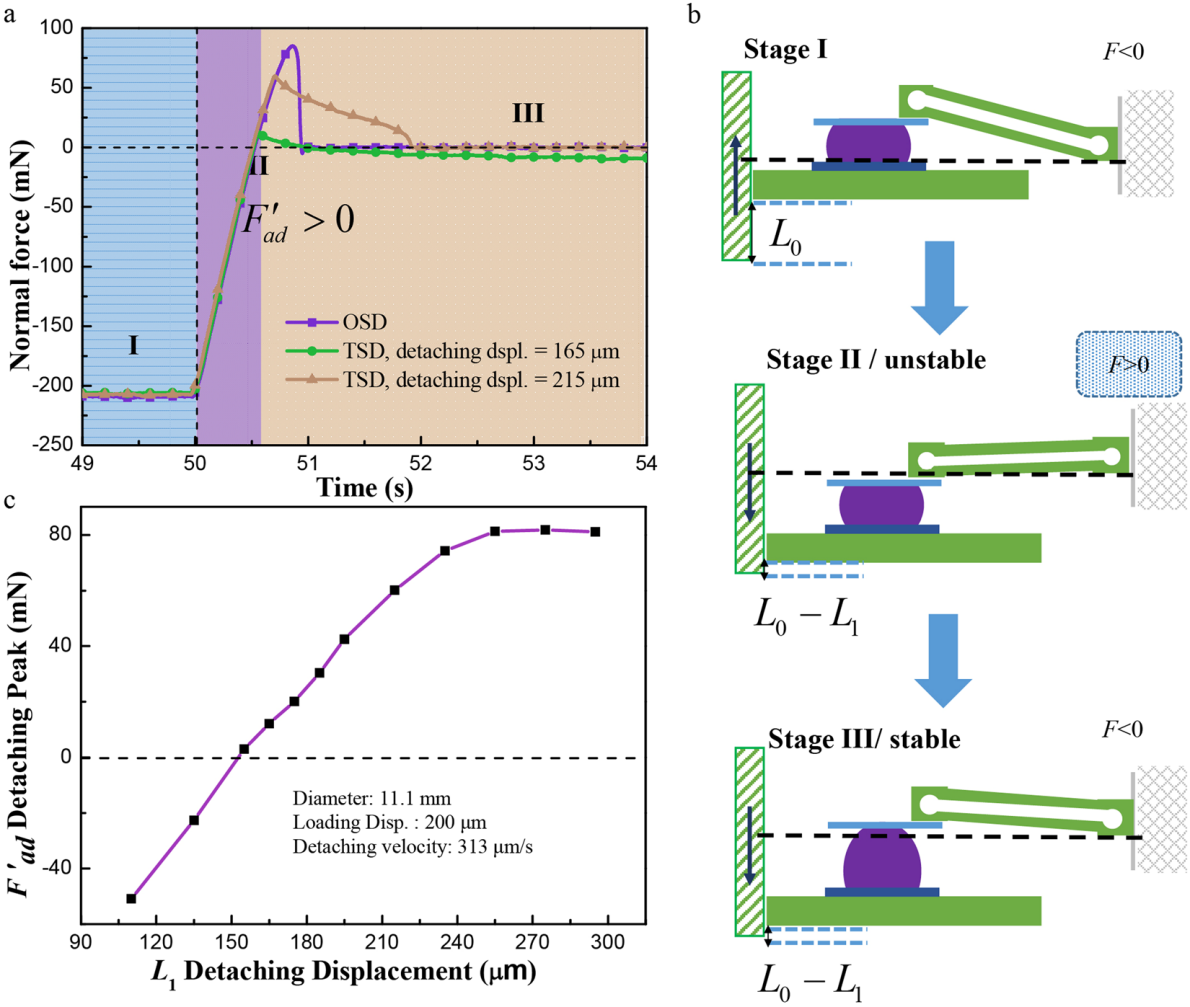
Procedure of the first detaching peak in TSD. (**a**) Normal force vs. time curve of OSD and TSD for a sphere diameter of 11.1 mm, the detaching velocity of 313 μm/s, *L*_0_ = 200 μm, *L*_1_ = 165 or 215 μm; (**b**) Sketch of the deformation procedure around the transient adhesion force peak; (**c**) *F′*_ad_ vs. detaching displacement for a sphere diameter of 11.1 mm, the detaching velocity of 313 μm/s and a loading displacement of 200 μm.

When the first detaching displacement was larger than a certain value, *F′*_ad_ in the TSD curve was positive, indicating an adhesive force, as shown in Fig. 2a. Then the contact was relaxed and the force can be turned into negative and repulsive again. This adhesion, when the applied unloading displacement is obviously below the loading displacement, is called transient adhesion force in this study.

When the sphere was loaded in Stage I, the double-cantilever was bended upward to press the sphere, as shown in Fig. 2b. Then, the sphere was moved down as the plate (in blue) followed the moving in Stage II. The interfacial viscoelasticity of the contact hindered the crack propagation, and the normal force was positive and adhesive. The contact reached a stable state (Stage III) after some time, and the force was negative and repulsive again. As shown in Fig. 2c, *F′*_ad_ increased with *L*_1_. After *L*_1_ reached the displacement where the max adhesion force was achieved, *F′*_ad_ became saturated. *F′*_ad_ for the experiment condition of the sphere with a radius 11.1 mm, the detaching velocity of 313 μm/s, *L*_0_ = 200 μm, became positive at the critical point *L′*_1_* = *155 μm. In fact, the first detachment is a cut-off in the full detachment, as shown in Fig. 2a. So the value of *L′*_1_, affected by the interfacial viscoelasticity of sphere-plate contact and the detaching velocity, is related to the zero-force point in the full detachment. Based on the diagram in Fig. 2b, the deformations in this adhesion measuring system should satisfy

1 $$\delta +{{\rm{\Delta }}}_{1}={L}_{0}-{L}_{1},$$

where *δ* is the deformation displacement of the sphere and Δ_1_ is the deflection of the cantilever. However, the brown line in Fig. 2a indicated another detaching situation. In this situation, the detaching displacement (215 μm) was beyond the loading displacement, whereas the contact was not fully separated before the stop of the motion. The measured adhesion force was obviously smaller than that in OSD. The mechanism of this phenomenon is the same as that of the transient adhesion. Furthermore, this adhesion might be mistakenly taken as the fully-detached adhesion in a real adhesion test because the contact was fully broken after the relaxation, similar to that in OSD.

### Effect of the elastic modulus

**Figure 3:**
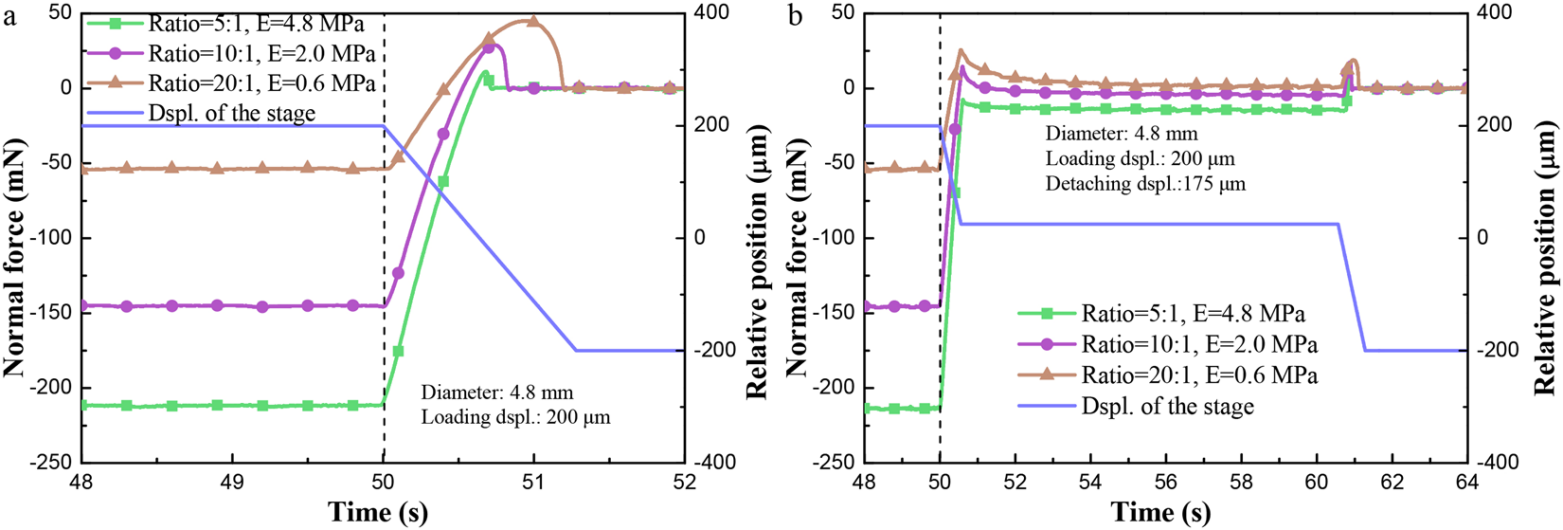
Force–time curves in OSD and TSD for PDMS sphere made of ratio 5:1, 10:1 and 20:1, respectively. (**a**) Curve in OSD, a loading displacement of 200 μm with different PDMS ratios (diameter *D* = 4.8 mm, detaching velocity *v* = 313 μm/s); (**b**) Curve in TSD, first detaching displacement of 175 μm and a loading displacement of 200 μm with different PDMS ratios (diameter *D* = 4.8 mm, detaching velocity *v* = 313 μm/s).

The effect of the elastic modulus on adhesion forces was experimentally studied. The PDMS sphere in A/B component ratio of 5:1 (*E* = 4.8 MPa) indicates the largest Young’s modulus compared with that in ratio 10:1 (*E* = 2.0 MPa) and 20:1 (*E* = 0.6 MPa). As shown in Fig. 3a, the sphere with a smaller Young’s modulus led to a larger *F′*_ad_. In fact, the tested adhesion force is the result of competition between the surface force and the elastic force. Thus with a smaller elastic force that results from a smaller Young’s modulus, the adhesion force increased, as shown in Fig. 3a. The similar result for TSD was shown in Fig. 3b that *F′*_ad_ and *F″*_ad_ followed the same trend.

### Effect of the cantilever stiffness

**Figure 4:**
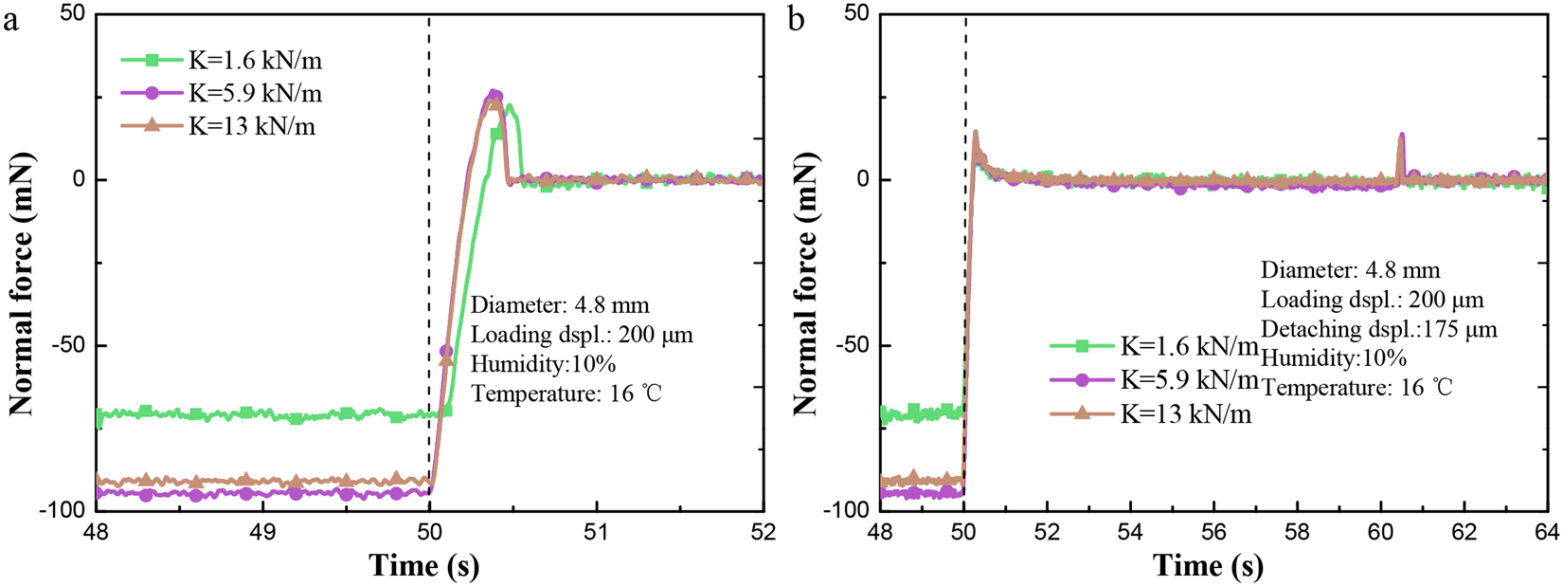
Force–time curves in OSD and TSD for different cantilever stiffness of 1.6, 5.9, 13 kN/m, respectively, with the humidity of 10% and environment temperature of 16 degrees Celsius. (**a**) Curve in OSD and a loading displacement of 200 μm with different cantilever stiffness (diameter *D* = 4.8 mm, detaching velocity *v* = 625 μm/s); (**b**) Curve in TSD, first detaching displacement of 175 μm and a loading displacement of 200 μm with different cantilever stiffness (diameter *D* = 4.8 mm, detaching velocity *v* = 625 μm/s).

As the elastic modulus of the sphere had a great effect on the adhesion forces, the effect of the cantilever stiffness was also studied. As shown in Fig. 4, *F′*_ad_ and *F*_ad_ with a larger cantilever stiffness were slightly larger due to the comparably larger peeling velocity at the edge of the contact area. Besides, compared with the stiffer cantilever, it took less time to reach the adhesion peak for the softer one in OSD.

### Effect of the detaching velocity

**Figure 5:**
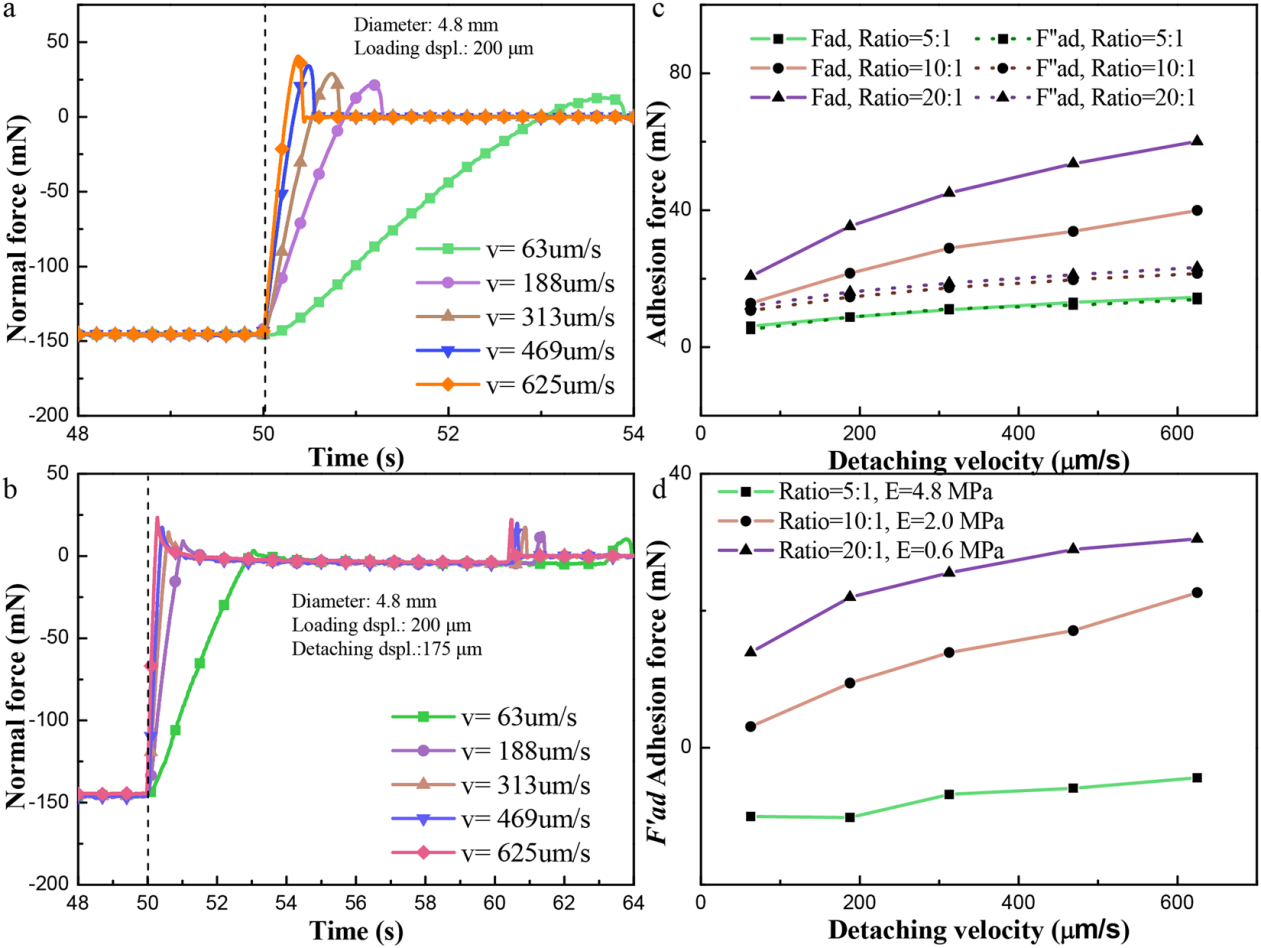
Normal force vs. time in OSD and TSD with different detaching velocities. (**a**) curve in OSD, totally detaching and a loading displacement of 200 μm at different detaching velocities *v* (diameter *D* = 4.8 mm); (**b**) curve in TSD, first detaching displacement of 175 μm and a loading displacement of 200 μm with different detaching velocities *v* (diameter *D* = 4.8 mm).

Experiments have also been conducted to research on the effects of detaching velocity. Results were shown in Fig. 5a and b. The adhesion force *F*_ad_ and *F″*_ad_ revealed a significant velocity effect and increased monotonously with the detaching velocity. The difference between *F*_ad_ and *F″*_ad_ in the same loading displacement and detaching velocity was not significant for the hardest sphere but were separated for the softer spheres, as shown in Fig. 5c. The change of *F′*_ad_ agreed with the velocity effect well (Fig. 5d). Furthermore, *F*_ad_ and *F″*_ad_ for the spheres with a larger Young’s modulus changed less along the increasing of detaching velocity. *F′*_ad_ also increased with the detaching velocity, as shown in Fig. 5d.

We switched the positions of sphere and plate (i.e., the sphere was fixed and the plate moved) to eliminate system error. With the same material, sphere diameter, detaching velocity and experimental procedure, *F*_ad_, *F′*_ad_ and *F″*_ad_ showed little difference.

## Theoretical analysis

**Figure 6:**
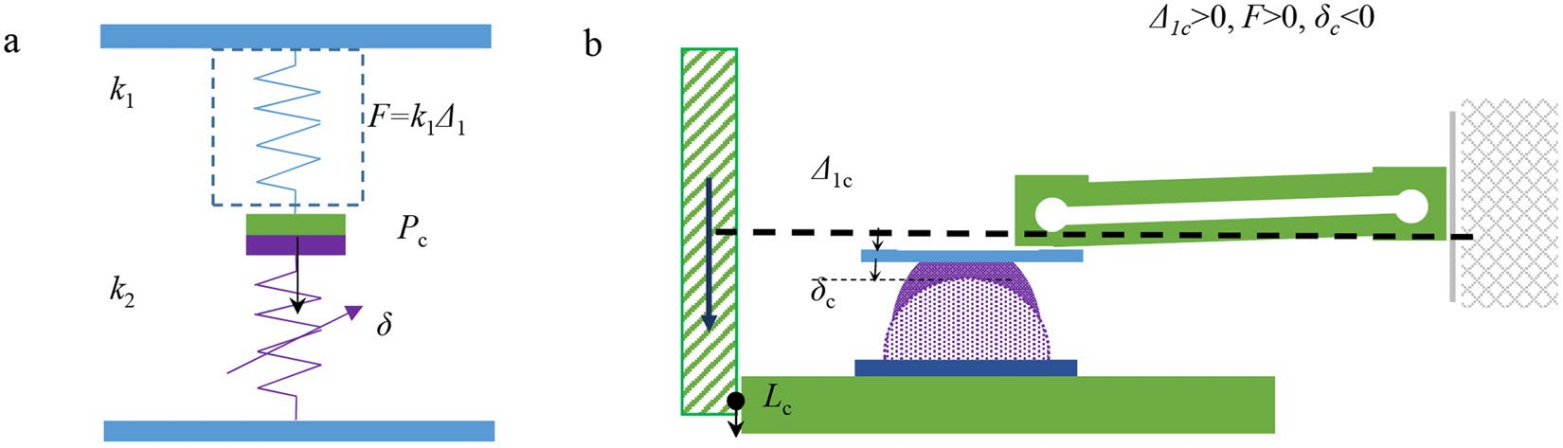
The spring model of the adhesion measuring system.

By neglecting the viscoelasticity of sphere and the deflection of the plate (PMMA is stiffer), the adhesion measuring system used in our experiments can be simplified as two springs in series bounded, with a fixed spring constant *k*_1_ for the double-cantilever and a displacement-dependent equivalent spring constant *k*_2_ for the sphere, as illustrated in Fig. 6a. The pull force *F*_c_, which equals the max adhesion force −*P*_c_ satisfies

2 $${F}_{c}={k}_{1}{{\rm{\Delta }}}_{1c}={k}_{2}{\delta }_{c}=-\,{P}_{c}=(3/2)\pi R\gamma ,$$

where *δ*_c_ and Δ_1c_ are the deformation displacement of sphere and deflection of the cantilever that correspond to the max adhesion force −*P*_c_, respectively.

Although the measured adhesion force *F*_c_ seems to be independent of the spring constant *k*_1_ based on Eq. (2), *k*_1_ can influence the shape of measured force-time curve^36^. The relationships between normal forces and displacements (or deflections) can be obtained by JKR model^37^ and Hooke’s law^38^, as follows:

3 $$\{\begin{array}{c}P={k}_{1}{{\rm{\Delta }}}_{1},\frac{\delta }{{\delta }_{c}}=(3\chi -1){(\frac{\chi +1}{9})}^{\frac{1}{3}}\\ \chi ={(\frac{P}{{P}_{c}}+1)}^{\frac{1}{2}},{\delta }_{c}=\frac{1}{3R}{(\frac{3R{P}_{c}}{K})}^{\frac{2}{3}},\end{array}$$

where 1/*K* = (1 − *v*_1_^2^)/*E*_1_ + (1 − *v*_2_^2^)/*E*_2_, *v* and *E* is the Poisson’s ratio and Young’s modulus for the materials of the sphere and plate, respectively. The signs of Δ_1_, *δ*, *L* and *F* were determined via Fig. 6b. Based on Eq. (1), the total displacement of the plate *L*_A_ from a loading displacement *L* to obtaining the max adhesion force can be calculated by

4 $${L}_{A}={L}_{0}-{L}_{1}-{L}_{c}={L}_{0}-{L}_{1}+\frac{3\pi R\gamma }{2{k}_{1}}-\frac{1}{3R}{(\frac{9\pi {R}^{2}\gamma }{2K})}^{\frac{2}{3}}.$$

*L*_A_ monotonically increases with the decreasing of *k*_1_. Given the same detaching velocity and loading displacement, more time is needed for attaining the max adhesion force for the cantilever with a smaller spring constant. The equivalent spring constant *k*_2_, determined by the elastic modulus of sphere, affects the measuring curve similarly. In Figs 3a and 4a, the sphere with a lower Young’s modulus or lower cantilever stiffness requires more time to reach the max adhesion force.

**Figure 7:**
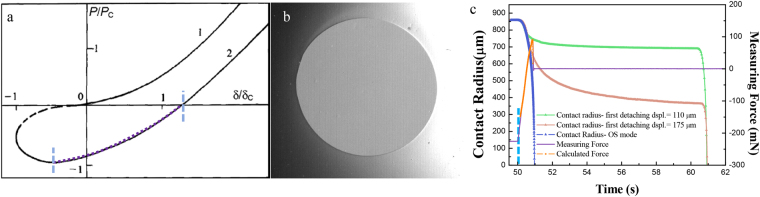
Calculation of the transient adhesion *F′*_ad_. (**a**) plot of *P/P*_c_ vs. *δ/δ*_c_ in Hertz (marked 1) and JKR theory (marked 2)^37^ (Permitted reprint from ‘Myshkin, N. K., Petrokovets, M. I. and Kovalev, A. V. *Tribol*. *Internat*. **38**, 910–21 (2005)’); (**b**) The CCD camera image of the contact area; (**c**) change of contact radius for three types of situations (OSD and TSD with first detaching displacement = 110 and 175 μm), measuring force during the one-step detachment and numerical fitting force-time curve.

We have presented that the value of the *F′*_ad_ in TSD may appear as a positive transient force. Theoretically, the value of transient adhesion in TSD sets on the JKR plot (OSD curve, in purple of Fig. 7a) where *P* = *F*_N_ < 0 but *P* does not reach −*P*_c_. Thus, this value can be estimated by using the modified JKR theory. Based on JKR theory, the normal force in OSD can be described as^10,39^

5 $${F}_{N}=\frac{4{E}^{\ast }{r}_{0}^{3}}{3R}-2{r}_{0}^{3/2}{(2\pi {E}^{\ast }{\gamma }_{eff})}^{1/2},$$

where E* = *K*, *r*_0_ is the radius of real contact area. *γ*_*eff*_ is different from the *γ* in JKR equation and represents the equivalent change of surface energy per contact area. *γ*_*eff*_ is strongly related with the crack velocity *v*_r_ (i.e., the change velocity of contact radius) and temperature *T*. At the same temperature *T*, the relationship *γ*_*eff*_ ~ (*v*_r_)^*α*^ has been obtained by researchers with *α* ≈ 0.6^9,11,40–42^, which was used in our calculation.

By analyzing pictures of contact zone (Fig. 7b), the differential of *r*_0_ to time (crack velocity *v*_r_) can be acquired from the *r*_0_ - *t* plot (Fig. 7c). Thus, we can use Eq. (5) to calculate *F′*_ad_. The fitting force-time curve in OSD was shown in Fig. 7c. The calculated results, for the PDMS sphere of diameter 11.1 mm with the detaching velocity of 313 μm/s and a loading displacement 200 μm, were shown in Table 1. The error between calculated *F*_cad_ (or *F′*_cad_) and measured *F*_ad_ (or *F′*_ad_) is given as Δ = |(*F*_cad_ − *F*_ad_)/*F*_ad_| × 100% (or Δ = |(*F′*_cad_ − *F′*_ad_)/*F′*_ad_| × 100%). Table 1 indicated that the calculation error was within an acceptable level. Contact radius at the transient peak in TSD (or the only peak in OSD) decreased as the increasing of the detaching displacement, also shown in Fig. 7c. A larger detaching displacement led to a higher loss of contact area along with the time.

**Table 1:** *F′*_ad_ (or *F*_ad_) and *F′*_cad_ (or *F*_cad_) for the PDMS sphere of diameter 11.1 mm with the detaching velocity of 313 μm/s and a loading displacement 200 μm.

	OSD	TSD	TSD	TSD	TSD
Detaching Dspl. *L*_1_/μm	—	100	130	190	220
Measured *F*_ad_/mN	85.5	—	—	—	—
Measured *F′*_ad_/mN	—	−78.8	−34.1	35.2	63.8
Calculated *F*_cad_ or *F′*_cad_/mN	91.0	−80.0	−36.5	39.0	63.7
Error Δ/%	6.4	1.5	7.0	10.8	0.2
Radius at *F′*_ad_ peak *r*/μm	358.8	783.6	749.2	640.1	564.2

According to the analysis, this transient adhesion would be more likely to appear in the adhesion tests with soft materials such as some polymers (PDMS, PU and so on) and hydrogels where we may need to concern about the effect of deformation compatibility of the cantilever measuring system. The short of detaching displacement led to an incomplete detachment, caused a loss of adhesion (see the brown line shown in the Fig. 2a), where it also appeared to be fully detached at the end. Thus it has a meaning in general adhesion tests. Besides, for the instruments such as atomic force microscopy (AFM)^43^ and surface force apparatus (SFA)^44^, the selection of the cantilever significantly affects the measuring results. To measure forces at the micro/nanoscale (molecular^45^ and cell interaction^43^ forces) with AFM, the softer cantilevers should be selected for accurately measuring the tip-sample interaction. However, the mechanical instability of the cantilever would lead to a jump-to-contact in the approaching procedure when the force gradient becomes larger than the spring constant of the free cantilever^45–47^. This jump shortens the measuring distance; however, the stiffer cantilevers can eliminate this jump but sacrifice the sensitivity^46^. In the adhesion test of gecko setae, the adhesion force may not be detected if the spring constant is not properly chosen^35^. On the other hand, the transient adhesion intrinsically results from interfacial viscoelasticity, which also leads to the adhesion hysteresis^47^ (the difference between the work needed to separate two surfaces and that to bring them together) in adhesion tests. For the silicone elastomers, the entanglement between the dangling chains on the surface mainly contributes to this contact hysteresis^48^ that researchers chemically treated (hydrolyzed^49^ or extracted^50^) the PDMS surface to investigate the effect of this mechanism on the adhesion. Thus, the study of the transient adhesion aids in complementarily understanding the cantilever-compatibility problem and the adhesion hysteresis in the adhesion tests.

## Conclusions

We found the transient adhesion in a non-full detachment of an adhesive contact. The detaching procedure is the compatibility among the sphere deformation, cantilever deflection, and stage movement. The origin of this transient adhesive force was ascribed to the recovery delay because of the crack propagation of the interface and the interfacial viscoelasticity of the contact. The transient adhesive force can be calculated by using a viscoelastic-modified JKR theory, and the fitting curve well agreed with the experimental one. These results disclosed that adhesion tests can be significantly affected by the stiffness of contact objects and the supporting spring cantilever, and that such tests should be carefully designed. All these results elucidated the nature of adhesion and can guide the design of adhesive tests and their applications.
